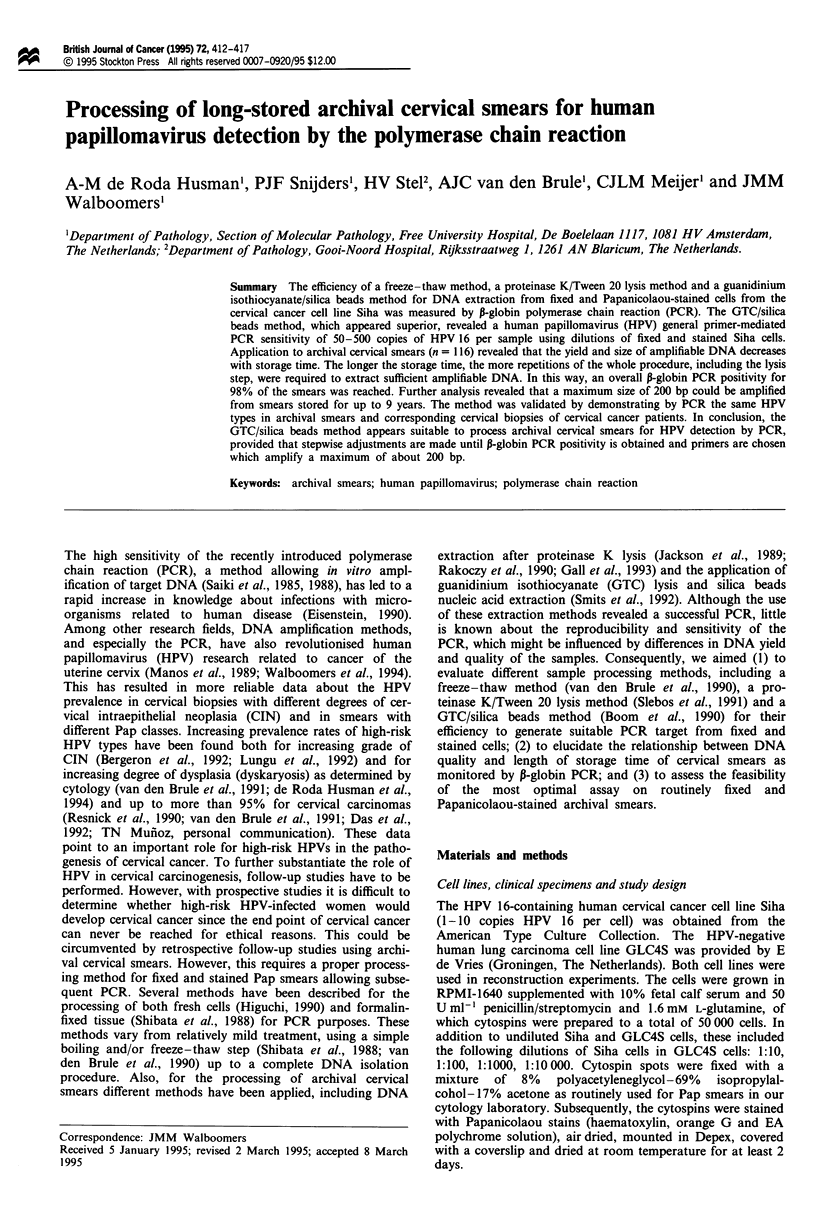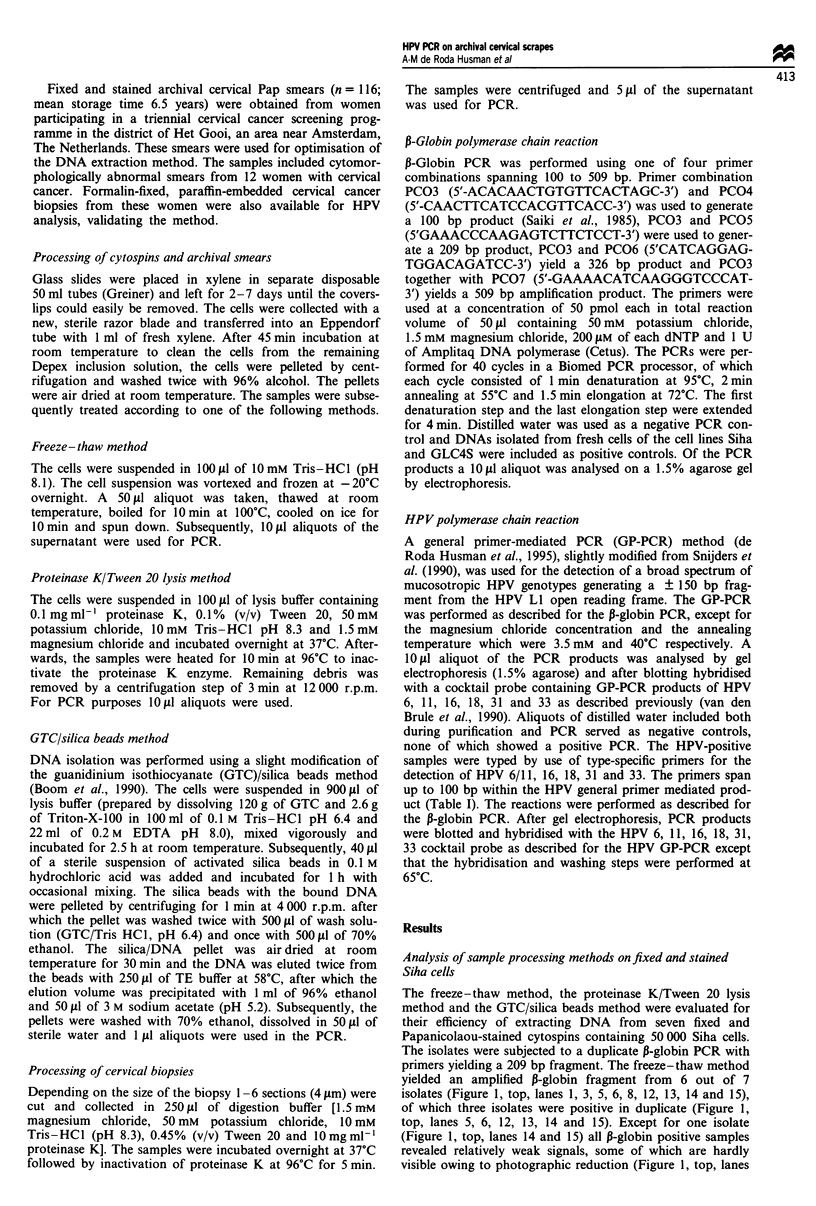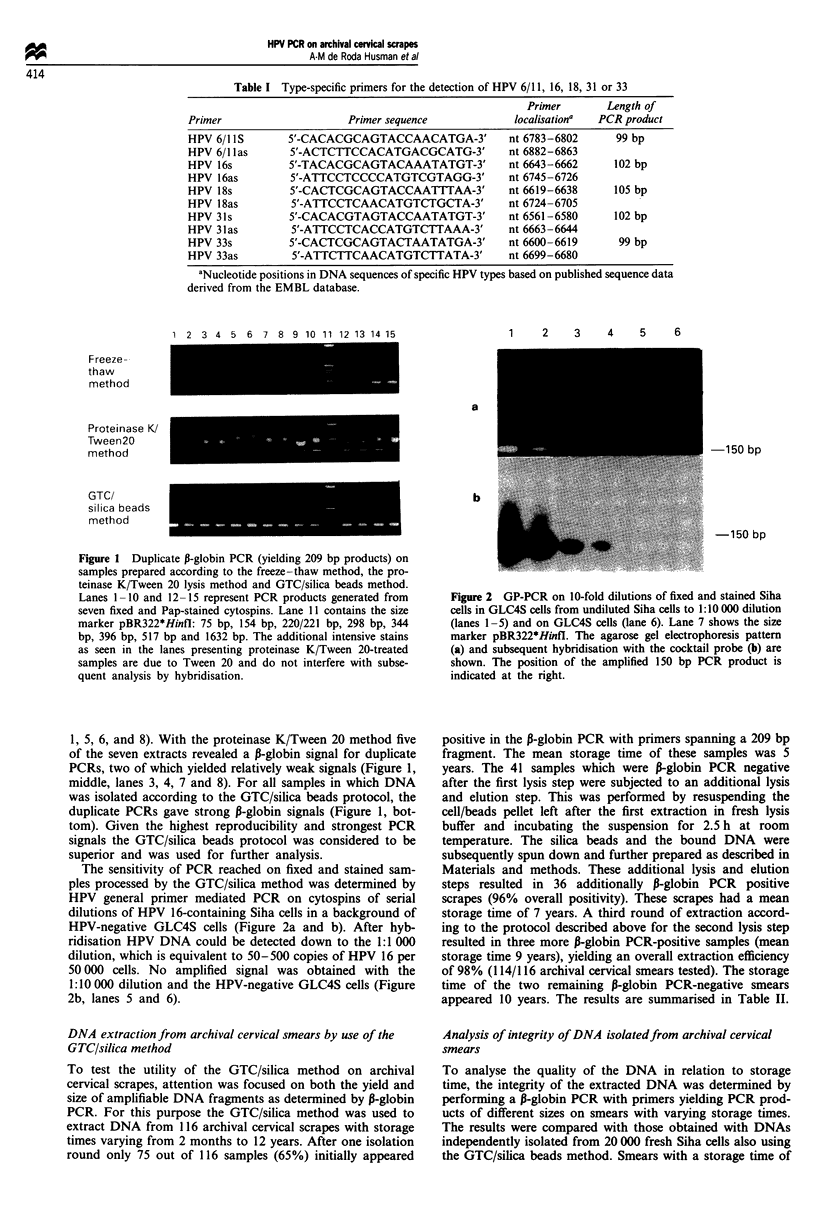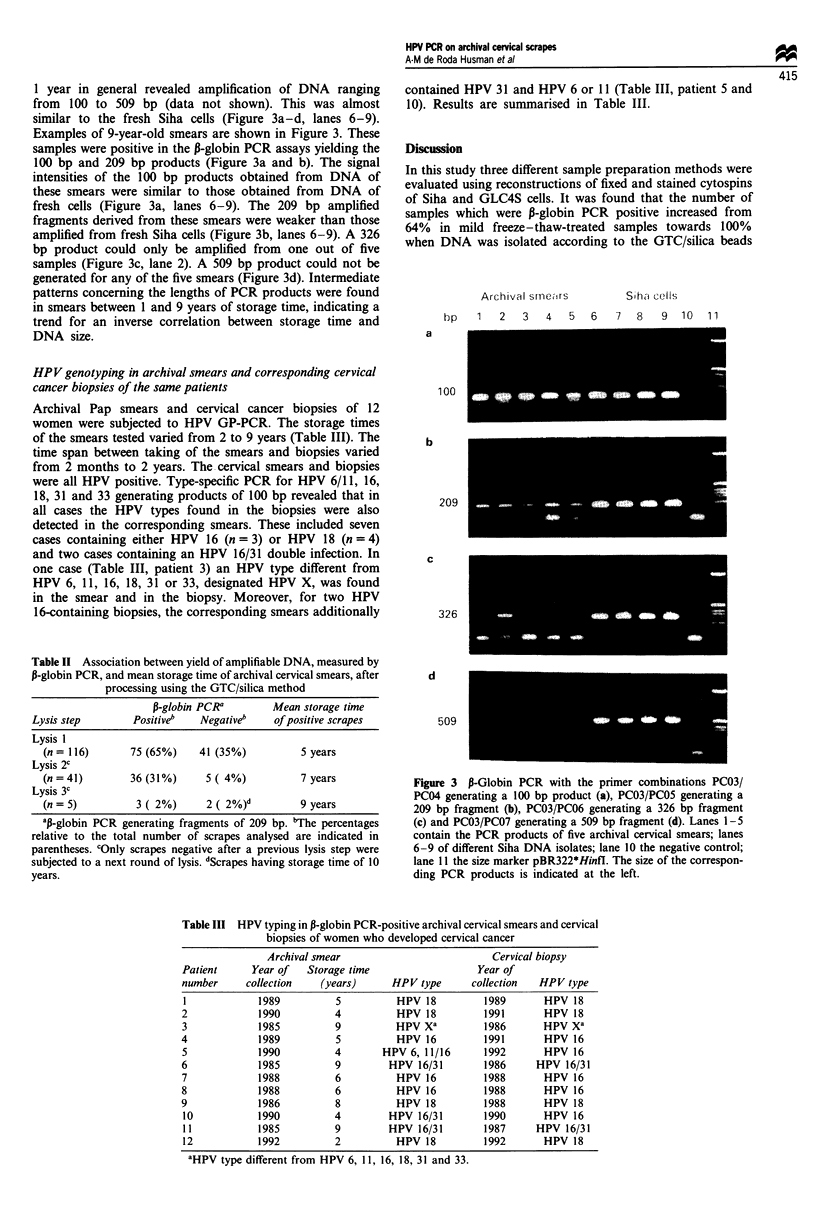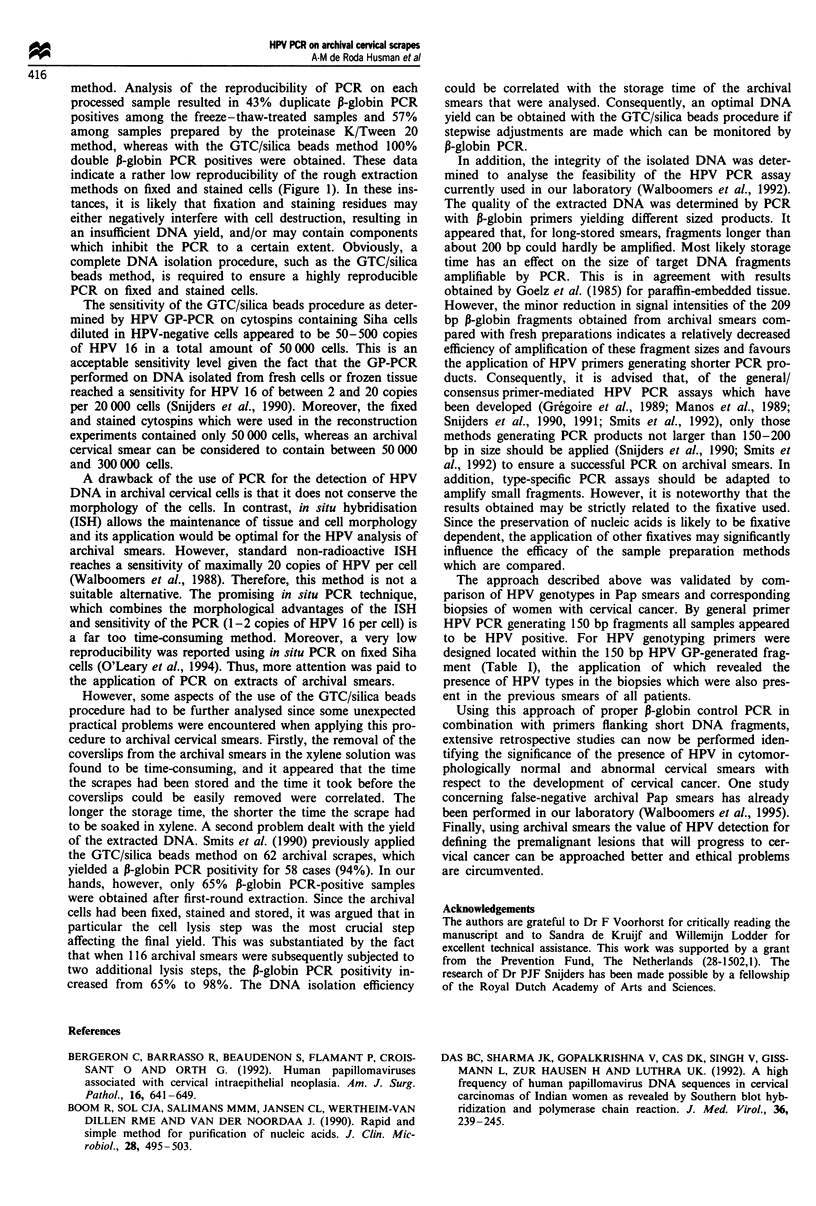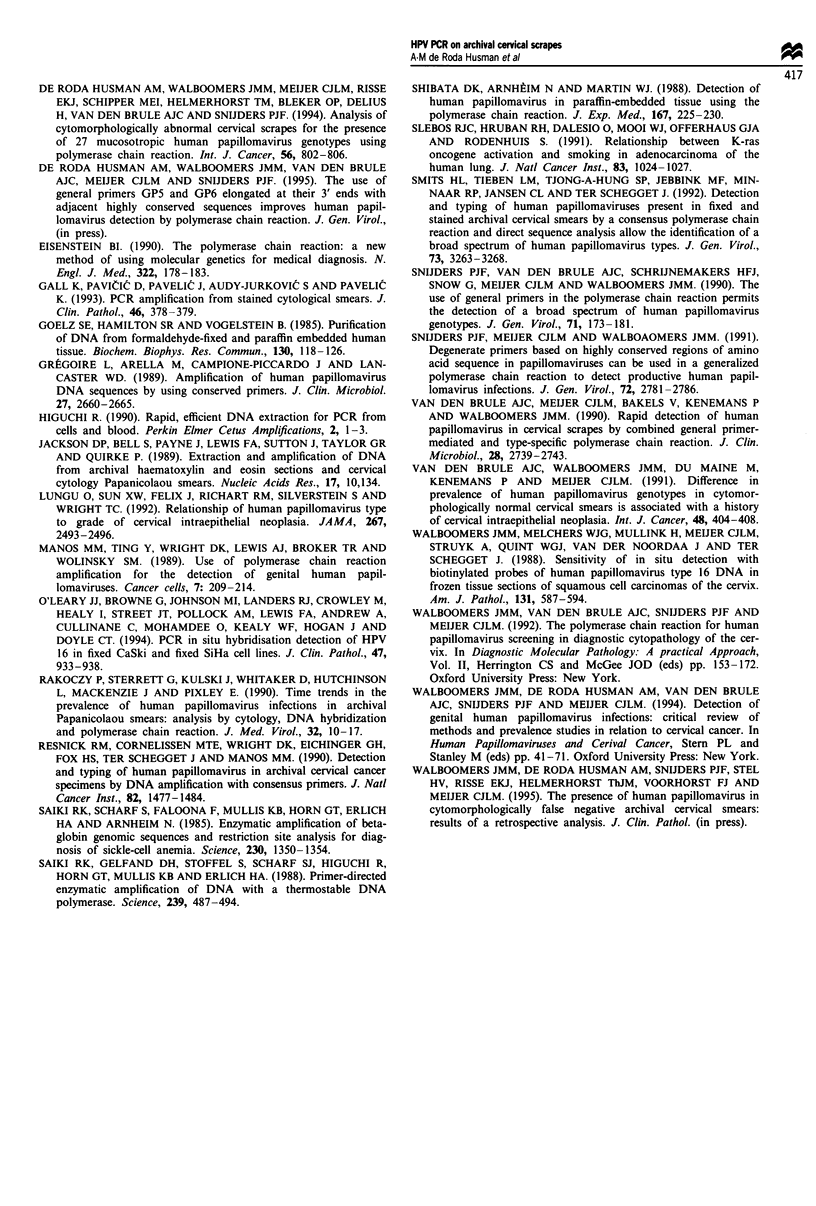# Processing of long-stored archival cervical smears for human papillomavirus detection by the polymerase chain reaction.

**DOI:** 10.1038/bjc.1995.347

**Published:** 1995-08

**Authors:** A. M. de Roda Husman, P. J. Snijders, H. V. Stel, A. J. van den Brule, C. J. Meijer, J. M. Walboomers

**Affiliations:** Department of Pathology, Free University Hospital, Amsterdam, The Netherlands.

## Abstract

**Images:**


British Journal of Cancer (1995) 72, 412-417
© 1995 Stockton Press All rights reserved 0007-0920/95 $12.00

### Processing of long-stored archival cervical smears for human papillomavirus detection by the polymerase chain reaction

A-M de Roda Husman[1], PJF Snijders[1], HV Stel[2], AJC van den Brule[1], CJLM Meijer[1] and JMM Walboomers[1]

[1]*Department of Pathology, Section of Molecular Pathology, Free University Hospital, De Boelelaan 1117, 1081 HV Amsterdam, The Netherlands;* [2]*Department of Pathology, Gooi-Noord Hospital, Rijksstraatweg 1, 1261 AN Blaricum, The Netherlands.*

**Summary** The efficiency of a freeze-thaw method, a proteinase K/Tween 20 lysis method and a guanidinium isothiocyanate/silica beads method for DNA extraction from fixed and Papanicolaou-stained cells from the cervical cancer cell line Siha was measured by β-globin polymerase chain reaction (PCR). The GTC/silica beads method, which appeared superior, revealed a human papillomavirus (HPV) general primer-mediated PCR sensitivity of 50-500 copies of HPV 16 per sample using dilutions of fixed and stained Siha cells. Application to archival cervical smears ($n$ = 116) revealed that the yield and size of amplifiable DNA decreases with storage time. The longer the storage time, the more repetitions of the whole procedure, including the lysis step, were required to extract sufficient amplifiable DNA. In this way, an overall β-globin PCR positivity for 98% of the smears was reached. Further analysis revealed that a maximum size of 200 bp could be amplified from smears stored for up to 9 years. The method was validated by demonstrating by PCR the same HPV types in archival smears and corresponding cervical biopsies of cervical cancer patients. In conclusion, the GTC/silica beads method appears suitable to process archival cervical smears for HPV detection by PCR, provided that stepwise adjustments are made until β-globin PCR positivity is obtained and primers are chosen which amplify a maximum of about 200 bp.

**Keywords:** archival smears; human papillomavirus; polymerase chain reaction

The high sensitivity of the recently introduced polymerase chain reaction (PCR), a method allowing *in vitro* amplification of target DNA (Saiki *et al.*, 1985, 1988), has led to a rapid increase in knowledge about infections with microorganisms related to human disease (Eisenstein, 1990). Among other research fields, DNA amplification methods, and especially the PCR, have also revolutionised human papillomavirus (HPV) research related to cancer of the uterine cervix (Manos *et al.*, 1989; Walboomers *et al.*, 1994). This has resulted in more reliable data about the HPV prevalence in cervical biopsies with different degrees of cervical intraepithelial neoplasia (CIN) and in smears with different Pap classes. Increasing prevalence rates of high-risk HPV types have been found both for increasing grade of CIN (Bergeron *et al.*, 1992; Lungu *et al.*, 1992) and for increasing degree of dysplasia (dyskaryosis) as determined by cytology (van den Brule *et al.*, 1991; de Roda Husman *et al.*, 1994) and up to more than 95% for cervical carcinomas (Resnick *et al.*, 1990; van den Brule *et al.*, 1991; Das *et al.*, 1992; TN Muñoz, personal communication). These data point to an important role for high-risk HPVs in the pathogenesis of cervical cancer. To further substantiate the role of HPV in cervical carcinogenesis, follow-up studies have to be performed. However, with prospective studies it is difficult to determine whether high-risk HPV-infected women would develop cervical cancer since the end point of cervical cancer can never be reached for ethical reasons. This could be circumvented by retrospective follow-up studies using archival cervical smears. However, this requires a proper processing method for fixed and stained Pap smears allowing subsequent PCR. Several methods have been described for the processing of both fresh cells (Higuchi, 1990) and formalin-fixed tissue (Shibata *et al.*, 1988) for PCR purposes. These methods vary from relatively mild treatment, using a simple boiling and/or freeze-thaw step (Shibata *et al.*, 1988; van den Brule *et al.*, 1990) up to a complete DNA isolation procedure. Also, for the processing of archival cervical smears different methods have been applied, including DNA extraction after proteinase K lysis (Jackson *et al.*, 1989; Rakoczy *et al.*, 1990; Gall *et al.*, 1993) and the application of guanidinium isothiocyanate (GTC) lysis and silica beads nucleic acid extraction (Smits *et al.*, 1992). Although the use of these extraction methods revealed a successful PCR, little is known about the reproducibility and sensitivity of the PCR, which might be influenced by differences in DNA yield and quality of the samples. Consequently, we aimed (1) to evaluate different sample processing methods, including a freeze-thaw method (van den Brule *et al.*, 1990), a proteinase K/Tween 20 lysis method (Slebos *et al.*, 1991) and a GTC/silica beads method (Boom *et al.*, 1990) for their efficiency to generate suitable PCR target from fixed and stained cells; (2) to elucidate the relationship between DNA quality and length of storage time of cervical smears as monitored by β-globin PCR; and (3) to assess the feasibility of the most optimal assay on routinely fixed and Papanicolaou-stained archival smears.

Correspondence: JMM Walboomers
Received 5 January 1995; revised 2 March 1995; accepted 8 March 1995

### Materials and methods

### *Cell lines, clinical specimens and study design*

The HPV 16-containing human cervical cancer cell line Siha (1-10 copies HPV 16 per cell) was obtained from the American Type Culture Collection. The HPV-negative human lung carcinoma cell line GLC4S was provided by E de Vries (Groningen, The Netherlands). Both cell lines were used in reconstruction experiments. The cells were grown in RPMI-1640 supplemented with 10% fetal calf serum and 50 U $ml^{-1}$ penicillin/streptomycin and 1.6 mM L-glutamine, of which cytospins were prepared to a total of 50 000 cells. In addition to undiluted Siha and GLC4S cells, these included the following dilutions of Siha cells in GLC4S cells: 1:10, 1:100, 1:1000, 1:10 000. Cytospin spots were fixed with a mixture of 8% polyacetyleneglycol-69% isopropylalcohol-17% acetone as routinely used for Pap smears in our cytology laboratory. Subsequently, the cytospins were stained with Papanicolaou stains (haematoxylin, orange G and EA polychrome solution), air dried, mounted in Depex, covered with a coverslip and dried at room temperature for at least 2 days.

Fixed and stained archival cervical Pap smears ($n = 116$; mean storage time 6.5 years) were obtained from women participating in a triennial cervical cancer screening programme in the district of Het Gooi, an area near Amsterdam, The Netherlands. These smears were used for optimisation of the DNA extraction method. The samples included cytomorphologically abnormal smears from 12 women with cervical cancer. Formalin-fixed, paraffin-embedded cervical cancer biopsies from these women were also available for HPV analysis, validating the method.

*Processing of cytospins and archival smears*

Glass slides were placed in xylene in separate disposable 50 ml tubes (Greiner) and left for 2–7 days until the coverslips could easily be removed. The cells were collected with a new, sterile razor blade and transferred into an Eppendorf tube with 1 ml of fresh xylene. After 45 min incubation at room temperature to clean the cells from the remaining Depex inclusion solution, the cells were pelleted by centrifugation and washed twice with 96% alcohol. The pellets were air dried at room temperature. The samples were subsequently treated according to one of the following methods.

*Freeze–thaw method*

The cells were suspended in 100 μl of 10 mM Tris–HCl (pH 8.1). The cell suspension was vortexed and frozen at −20°C overnight. A 50 μl aliquot was taken, thawed at room temperature, boiled for 10 min at 100°C, cooled on ice for 10 min and spun down. Subsequently, 10 μl aliquots of the supernatant were used for PCR.

*Proteinase K/Tween 20 lysis method*

The cells were suspended in 100 μl of lysis buffer containing 0.1 mg ml$^{-1}$ proteinase K, 0.1% (v/v) Tween 20, 50 mM potassium chloride, 10 mM Tris–HCl pH 8.3 and 1.5 mM magnesium chloride and incubated overnight at 37°C. Afterwards, the samples were heated for 10 min at 96°C to inactivate the proteinase K enzyme. Remaining debris was removed by a centrifugation step of 3 min at 12 000 r.p.m. For PCR purposes 10 μl aliquots were used.

*GTC/silica beads method*

DNA isolation was performed using a slight modification of the guanidinium isothiocyanate (GTC)/silica beads method (Boom *et al.*, 1990). The cells were suspended in 900 μl of lysis buffer (prepared by dissolving 120 g of GTC and 2.6 g of Triton-X-100 in 100 ml of 0.1 M Tris–HCl pH 6.4 and 22 ml of 0.2 M EDTA pH 8.0), mixed vigorously and incubated for 2.5 h at room temperature. Subsequently, 40 μl of a sterile suspension of activated silica beads in 0.1 M hydrochloric acid was added and incubated for 1 h with occasional mixing. The silica beads with the bound DNA were pelleted by centrifuging for 1 min at 4 000 r.p.m. after which the pellet was washed twice with 500 μl of wash solution (GTC/Tris HCl, pH 6.4) and once with 500 μl of 70% ethanol. The silica/DNA pellet was air dried at room temperature for 30 min and the DNA was eluted twice from the beads with 250 μl of TE buffer at 58°C, after which the elution volume was precipitated with 1 ml of 96% ethanol and 50 μl of 3 M sodium acetate (pH 5.2). Subsequently, the pellets were washed with 70% ethanol, dissolved in 50 μl of sterile water and 1 μl aliquots were used in the PCR.

*Processing of cervical biopsies*

Depending on the size of the biopsy 1–6 sections (4 μm) were cut and collected in 250 μl of digestion buffer [1.5 mM magnesium chloride, 50 mM potassium chloride, 10 mM Tris–HCl (pH 8.3), 0.45% (v/v) Tween 20 and 10 mg ml$^{-1}$ proteinase K]. The samples were incubated overnight at 37°C followed by inactivation of proteinase K at 96°C for 5 min. The samples were centrifuged and 5 μl of the supernatant was used for PCR.

*β-Globin polymerase chain reaction*

β-Globin PCR was performed using one of four primer combinations spanning 100 to 509 bp. Primer combination PCO3 (5′-ACACAACTGTGTTCACTAGC-3′) and PCO4 (5′-CAACTTCATCCACGTTCACC-3′) was used to generate a 100 bp product (Saiki *et al.*, 1985), PCO3 and PCO5 (5′GAAACCCAAGAGTCTTCTCCT-3′) were used to generate a 209 bp product, PCO3 and PCO6 (5′CATCAGGAGTGGACAGATCC-3′) yield a 326 bp product and PCO3 together with PCO7 (5′-GAAAACATCAAGGGTCCCAT-3′) yields a 509 bp amplification product. The primers were used at a concentration of 50 pmol each in total reaction volume of 50 μl containing 50 mM potassium chloride, 1.5 mM magnesium chloride, 200 μM of each dNTP and 1 U of Amplitaq DNA polymerase (Cetus). The PCRs were performed for 40 cycles in a Biomed PCR processor, of which each cycle consisted of 1 min denaturation at 95°C, 2 min annealing at 55°C and 1.5 min elongation at 72°C. The first denaturation step and the last elongation step were extended for 4 min. Distilled water was used as a negative PCR control and DNAs isolated from fresh cells of the cell lines Siha and GLC4S were included as positive controls. Of the PCR products a 10 μl aliquot was analysed on a 1.5% agarose gel by electrophoresis.

*HPV polymerase chain reaction*

A general primer-mediated PCR (GP-PCR) method (de Roda Husman *et al.*, 1995), slightly modified from Snijders *et al.* (1990), was used for the detection of a broad spectrum of mucosotropic HPV genotypes generating a ± 150 bp fragment from the HPV L1 open reading frame. The GP-PCR was performed as described for the β-globin PCR, except for the magnesium chloride concentration and the annealing temperature which were 3.5 mM and 40°C respectively. A 10 μl aliquot of the PCR products was analysed by gel electrophoresis (1.5% agarose) and after blotting hybridised with a cocktail probe containing GP-PCR products of HPV 6, 11, 16, 18, 31 and 33 as described previously (van den Brule *et al.*, 1990). Aliquots of distilled water included both during purification and PCR served as negative controls, none of which showed a positive PCR. The HPV-positive samples were typed by use of type-specific primers for the detection of HPV 6/11, 16, 18, 31 and 33. The primers span up to 100 bp within the HPV general primer mediated product (Table I). The reactions were performed as described for the β-globin PCR. After gel electrophoresis, PCR products were blotted and hybridised with the HPV 6, 11, 16, 18, 31, 33 cocktail probe as described for the HPV GP-PCR except that the hybridisation and washing steps were performed at 65°C.

### Results

*Analysis of sample processing methods on fixed and stained Siha cells*

The freeze–thaw method, the proteinase K/Tween 20 lysis method and the GTC/silica beads method were evaluated for their efficiency of extracting DNA from seven fixed and Papanicolaou-stained cytospins containing 50 000 Siha cells. The isolates were subjected to a duplicate β-globin PCR with primers yielding a 209 bp fragment. The freeze–thaw method yielded an amplified β-globin fragment from 6 out of 7 isolates (Figure 1, top, lanes 1, 3, 5, 6, 8, 12, 13, 14 and 15), of which three isolates were positive in duplicate (Figure 1, top, lanes 5, 6, 12, 13, 14 and 15). Except for one isolate (Figure 1, top, lanes 14 and 15) all β-globin positive samples revealed relatively weak signals, some of which are hardly visible owing to photographic reduction (Figure 1, top, lanes

**Table I** Type-specific primers for the detection of HPV 6/11, 16, 18, 31 or 33

| *Primer* | *Primer sequence* | *Primer localisation*[a] | *Length of PCR product* |
|---|---|---|---|
| HPV 6/11S | 5'-CACACGCAGTACCAACATGA-3' | nt 6783-6802 | 99 bp |
| HPV 6/11as | 5'-ACTCTTCCACATGACGCATG-3' | nt 6882-6863 | |
| HPV 16s | 5'-TACACGCAGTACAAATATGT-3' | nt 6643-6662 | 102 bp |
| HPV 16as | 5'-ATTCCTCCCCATGTCGTAGG-3' | nt 6745-6726 | |
| HPV 18s | 5'-CACTCGCAGTACCAATTTAA-3' | nt 6619-6638 | 105 bp |
| HPV 18as | 5'-ATTCCTCAACATGTCTGCTA-3' | nt 6724-6705 | |
| HPV 31s | 5'-CACACGTAGTACCAATATGT-3' | nt 6561-6580 | 102 bp |
| HPV 31as | 5'-ATTCCTCACCATGTCTTAAA-3' | nt 6663-6644 | |
| HPV 33s | 5'-CACTCGCAGTACTAATATGA-3' | nt 6600-6619 | 99 bp |
| HPV 33as | 5'-ATTCTTCAACATGTCTTATA-3' | nt 6699-6680 | |

[a]Nucleotide positions in DNA sequences of specific HPV types based on published sequence data derived from the EMBL database.

**Figure 1** Duplicate β-globin PCR (yielding 209 bp products) on samples prepared according to the freeze–thaw method, the proteinase K/Tween 20 lysis method and GTC/silica beads method. Lanes 1–10 and 12–15 represent PCR products generated from seven fixed and Pap-stained cytospins. Lane 11 contains the size marker pBR322**Hin*fI: 75 bp, 154 bp, 220/221 bp, 298 bp, 344 bp, 396 bp, 517 bp and 1632 bp. The additional intensive stains as seen in the lanes presenting proteinase K/Tween 20-treated samples are due to Tween 20 and do not interfere with subsequent analysis by hybridisation.

**Figure 2** GP-PCR on 10-fold dilutions of fixed and stained Siha cells in GLC4S cells from undiluted Siha cells to 1:10 000 dilution (lanes 1–5) and on GLC4S cells (lane 6). Lane 7 shows the size marker pBR322**Hin*fI. The agarose gel electrophoresis pattern (**a**) and subsequent hybridisation with the cocktail probe (**b**) are shown. The position of the amplified 150 bp PCR product is indicated at the right.

1, 5, 6, and 8). With the proteinase K/Tween 20 method five of the seven extracts revealed a β-globin signal for duplicate PCRs, two of which yielded relatively weak signals (Figure 1, middle, lanes 3, 4, 7 and 8). For all samples in which DNA was isolated according to the GTC/silica beads protocol, the duplicate PCRs gave strong β-globin signals (Figure 1, bottom). Given the highest reproducibility and strongest PCR signals the GTC/silica beads protocol was considered to be superior and was used for further analysis.

The sensitivity of PCR reached on fixed and stained samples processed by the GTC/silica method was determined by HPV general primer mediated PCR on cytospins of serial dilutions of HPV 16-containing Siha cells in a background of HPV-negative GLC4S cells (Figure 2a and b). After hybridisation HPV DNA could be detected down to the 1:1 000 dilution, which is equivalent to 50–500 copies of HPV 16 per 50 000 cells. No amplified signal was obtained with the 1:10 000 dilution and the HPV-negative GLC4S cells (Figure 2b, lanes 5 and 6).

*DNA extraction from archival cervical smears by use of the GTC/silica method*

To test the utility of the GTC/silica method on archival cervical scrapes, attention was focused on both the yield and size of amplifiable DNA fragments as determined by β-globin PCR. For this purpose the GTC/silica method was used to extract DNA from 116 archival cervical scrapes with storage times varying from 2 months to 12 years. After one isolation round only 75 out of 116 samples (65%) initially appeared positive in the β-globin PCR with primers spanning a 209 bp fragment. The mean storage time of these samples was 5 years. The 41 samples which were β-globin PCR negative after the first lysis step were subjected to an additional lysis and elution step. This was performed by resuspending the cell/beads pellet left after the first extraction in fresh lysis buffer and incubating the suspension for 2.5 h at room temperature. The silica beads and the bound DNA were subsequently spun down and further prepared as described in Materials and methods. These additional lysis and elution steps resulted in 36 additionally β-globin PCR positive scrapes (96% overall positivity). These scrapes had a mean storage time of 7 years. A third round of extraction according to the protocol described above for the second lysis step resulted in three more β-globin PCR-positive samples (mean storage time 9 years), yielding an overall extraction efficiency of 98% (114/116 archival cervical smears tested). The storage time of the two remaining β-globin PCR-negative smears appeared 10 years. The results are summarised in Table II.

*Analysis of integrity of DNA isolated from archival cervical smears*

To analyse the quality of the DNA in relation to storage time, the integrity of the extracted DNA was determined by performing a β-globin PCR with primers yielding PCR products of different sizes on smears with varying storage times. The results were compared with those obtained with DNAs independently isolated from 20 000 fresh Siha cells also using the GTC/silica beads method. Smears with a storage time of

1 year in general revealed amplification of DNA ranging from 100 to 509 bp (data not shown). This was almost similar to the fresh Siha cells (Figure 3a–d, lanes 6–9). Examples of 9-year-old smears are shown in Figure 3. These samples were positive in the β-globin PCR assays yielding the 100 bp and 209 bp products (Figure 3a and b). The signal intensities of the 100 bp products obtained from DNA of these smears were similar to those obtained from DNA of fresh cells (Figure 3a, lanes 6–9). The 209 bp amplified fragments derived from these smears were weaker than those amplified from fresh Siha cells (Figure 3b, lanes 6–9). A 326 bp product could only be amplified from one out of five samples (Figure 3c, lane 2). A 509 bp product could not be generated for any of the five smears (Figure 3d). Intermediate patterns concerning the lengths of PCR products were found in smears between 1 and 9 years of storage time, indicating a trend for an inverse correlation between storage time and DNA size.

### *HPV genotyping in archival smears and corresponding cervical cancer biopsies of the same patients*

Archival Pap smears and cervical cancer biopsies of 12 women were subjected to HPV GP-PCR. The storage times of the smears tested varied from 2 to 9 years (Table III). The time span between taking of the smears and biopsies varied from 2 months to 2 years. The cervical smears and biopsies were all HPV positive. Type-specific PCR for HPV 6/11, 16, 18, 31 and 33 generating products of 100 bp revealed that in all cases the HPV types found in the biopsies were also detected in the corresponding smears. These included seven cases containing either HPV 16 ($n = 3$) or HPV 18 ($n = 4$) and two cases containing an HPV 16/31 double infection. In one case (Table III, patient 3) an HPV type different from HPV 6, 11, 16, 18, 31 or 33, designated HPV X, was found in the smear and in the biopsy. Moreover, for two HPV 16-containing biopsies, the corresponding smears additionally contained HPV 31 and HPV 6 or 11 (Table III, patient 5 and 10). Results are summarised in Table III.

**Table II** Association between yield of amplifiable DNA, measured by β-globin PCR, and mean storage time of archival cervical smears, after processing using the GTC/silica method

| *Lysis step* | *β-globin PCR*[a] *Positive*[b] | *Negative*[b] | *Mean storage time of positive scrapes* |
|---|---|---|---|
| Lysis 1 ($n = 116$) | 75 (65%) | 41 (35%) | 5 years |
| Lysis 2[c] ($n = 41$) | 36 (31%) | 5 ( 4%) | 7 years |
| Lysis 3[c] ($n = 5$) | 3 ( 2%) | 2 ( 2%)[d] | 9 years |

[a]β-globin PCR generating fragments of 209 bp. [b]The percentages relative to the total number of scrapes analysed are indicated in parentheses. [c]Only scrapes negative after a previous lysis step were subjected to a next round of lysis. [d]Scrapes having storage time of 10 years.

### Discussion

In this study three different sample preparation methods were evaluated using reconstructions of fixed and stained cytospins of Siha and GLC4S cells. It was found that the number of samples which were β-globin PCR positive increased from 64% in mild freeze–thaw-treated samples towards 100% when DNA was isolated according to the GTC/silica beads

**Figure 3** β-Globin PCR with the primer combinations PC03/PC04 generating a 100 bp product (**a**), PC03/PC05 generating a 209 bp fragment (**b**), PC03/PC06 generating a 326 bp fragment (**c**) and PC03/PC07 generating a 509 bp fragment (**d**). Lanes 1–5 contain the PCR products of five archival cervical smears; lanes 6–9 of different Siha DNA isolates; lane 10 the negative control; lane 11 the size marker pBR322**Hin*fI. The size of the corresponding PCR products is indicated at the left.

**Table III** HPV typing in β-globin PCR-positive archival cervical smears and cervical biopsies of women who developed cervical cancer

| *Patient number* | *Archival smear* *Year of collection* | *Storage time (years)* | *HPV type* | *Cervical biopsy* *Year of collection* | *HPV type* |
|---|---|---|---|---|---|
| 1 | 1989 | 5 | HPV 18 | 1989 | HPV 18 |
| 2 | 1990 | 4 | HPV 18 | 1991 | HPV 18 |
| 3 | 1985 | 9 | HPV X[a] | 1986 | HPV X[a] |
| 4 | 1989 | 5 | HPV 16 | 1991 | HPV 16 |
| 5 | 1990 | 4 | HPV 6, 11/16 | 1992 | HPV 16 |
| 6 | 1985 | 9 | HPV 16/31 | 1986 | HPV 16/31 |
| 7 | 1988 | 6 | HPV 16 | 1988 | HPV 16 |
| 8 | 1988 | 6 | HPV 16 | 1988 | HPV 16 |
| 9 | 1986 | 8 | HPV 18 | 1988 | HPV 18 |
| 10 | 1990 | 4 | HPV 16/31 | 1990 | HPV 16 |
| 11 | 1985 | 9 | HPV 16/31 | 1987 | HPV 16/31 |
| 12 | 1992 | 2 | HPV 18 | 1992 | HPV 18 |

[a]HPV type different from HPV 6, 11, 16, 18, 31 and 33.

method. Analysis of the reproducibility of PCR on each processed sample resulted in 43% duplicate β-globin PCR positives among the freeze–thaw-treated samples and 57% among samples prepared by the proteinase K/Tween 20 method, whereas with the GTC/silica beads method 100% double β-globin PCR positives were obtained. These data indicate a rather low reproducibility of the rough extraction methods on fixed and stained cells (Figure 1). In these instances, it is likely that fixation and staining residues may either negatively interfere with cell destruction, resulting in an insufficient DNA yield, and/or may contain components which inhibit the PCR to a certain extent. Obviously, a complete DNA isolation procedure, such as the GTC/silica beads method, is required to ensure a highly reproducible PCR on fixed and stained cells.

The sensitivity of the GTC/silica beads procedure as determined by HPV GP-PCR on cytospins containing Siha cells diluted in HPV-negative cells appeared to be 50–500 copies of HPV 16 in a total amount of 50 000 cells. This is an acceptable sensitivity level given the fact that the GP-PCR performed on DNA isolated from fresh cells or frozen tissue reached a sensitivity for HPV 16 of between 2 and 20 copies per 20 000 cells (Snijders *et al.*, 1990). Moreover, the fixed and stained cytospins which were used in the reconstruction experiments contained only 50 000 cells, whereas an archival cervical smear can be considered to contain between 50 000 and 300 000 cells.

A drawback of the use of PCR for the detection of HPV DNA in archival cervical cells is that it does not conserve the morphology of the cells. In contrast, *in situ* hybridisation (ISH) allows the maintenance of tissue and cell morphology and its application would be optimal for the HPV analysis of archival smears. However, standard non-radioactive ISH reaches a sensitivity of maximally 20 copies of HPV per cell (Walboomers *et al.*, 1988). Therefore, this method is not a suitable alternative. The promising *in situ* PCR technique, which combines the morphological advantages of the ISH and sensitivity of the PCR (1–2 copies of HPV 16 per cell) is a far too time-consuming method. Moreover, a very low reproducibility was reported using *in situ* PCR on fixed Siha cells (O'Leary *et al.*, 1994). Thus, more attention was paid to the application of PCR on extracts of archival smears.

However, some aspects of the use of the GTC/silica beads procedure had to be further analysed since some unexpected practical problems were encountered when applying this procedure to archival cervical smears. Firstly, the removal of the coverslips from the archival smears in the xylene solution was found to be time-consuming, and it appeared that the time the scrapes had been stored and the time it took before the coverslips could be easily removed were correlated. The longer the storage time, the shorter the time the scrape had to be soaked in xylene. A second problem dealt with the yield of the extracted DNA. Smits *et al.* (1990) previously applied the GTC/silica beads method on 62 archival scrapes, which yielded a β-globin PCR positivity for 58 cases (94%). In our hands, however, only 65% β-globin PCR-positive samples were obtained after first-round extraction. Since the archival cells had been fixed, stained and stored, it was argued that in particular the cell lysis step was the most crucial step affecting the final yield. This was substantiated by the fact that when 116 archival smears were subsequently subjected to two additional lysis steps, the β-globin PCR positivity increased from 65% to 98%. The DNA isolation efficiency could be correlated with the storage time of the archival smears that were analysed. Consequently, an optimal DNA yield can be obtained with the GTC/silica beads procedure if stepwise adjustments are made which can be monitored by β-globin PCR.

In addition, the integrity of the isolated DNA was determined to analyse the feasibility of the HPV PCR assay currently used in our laboratory (Walboomers *et al.*, 1992). The quality of the extracted DNA was determined by PCR with β-globin primers yielding different sized products. It appeared that, for long-stored smears, fragments longer than about 200 bp could hardly be amplified. Most likely storage time has an effect on the size of target DNA fragments amplifiable by PCR. This is in agreement with results obtained by Goelz *et al.* (1985) for paraffin-embedded tissue. However, the minor reduction in signal intensities of the 209 bp β-globin fragments obtained from archival smears compared with fresh preparations indicates a relatively decreased efficiency of amplification of these fragment sizes and favours the application of HPV primers generating shorter PCR products. Consequently, it is advised that, of the general/consensus primer-mediated HPV PCR assays which have been developed (Grégoire *et al.*, 1989; Manos *et al.*, 1989; Snijders *et al.*, 1990, 1991; Smits *et al.*, 1992), only those methods generating PCR products not larger than 150–200 bp in size should be applied (Snijders *et al.*, 1990; Smits *et al.*, 1992) to ensure a successful PCR on archival smears. In addition, type-specific PCR assays should be adapted to amplify small fragments. However, it is noteworthy that the results obtained may be strictly related to the fixative used. Since the preservation of nucleic acids is likely to be fixative dependent, the application of other fixatives may significantly influence the efficacy of the sample preparation methods which are compared.

The approach described above was validated by comparison of HPV genotypes in Pap smears and corresponding biopsies of women with cervical cancer. By general primer HPV PCR generating 150 bp fragments all samples appeared to be HPV positive. For HPV genotyping primers were designed located within the 150 bp HPV GP-generated fragment (Table I), the application of which revealed the presence of HPV types in the biopsies which were also present in the previous smears of all patients.

Using this approach of proper β-globin control PCR in combination with primers flanking short DNA fragments, extensive retrospective studies can now be performed identifying the significance of the presence of HPV in cytomorphologically normal and abnormal cervical smears with respect to the development of cervical cancer. One study concerning false-negative archival Pap smears has already been performed in our laboratory (Walboomers *et al.*, 1995). Finally, using archival smears the value of HPV detection for defining the premalignant lesions that will progress to cervical cancer can be approached better and ethical problems are circumvented.

**Acknowledgements**

The authors are grateful to Dr F Voorhorst for critically reading the manuscript and to Sandra de Kruijf and Willemijn Lodder for excellent technical assistance. This work was supported by a grant from the Prevention Fund, The Netherlands (28-1502,1). The research of Dr PJF Snijders has been made possible by a fellowship of the Royal Dutch Academy of Arts and Sciences.

### References

BERGERON C, BARRASSO R, BEAUDENON S, FLAMANT P, CROISSANT O AND ORTH G. (1992). Human papillomaviruses associated with cervical intraepithelial neoplasia. *Am. J. Surg. Pathol.*, **16**, 641–649.

BOOM R, SOL CJA, SALIMANS MMM, JANSEN CL, WERTHEIM-VAN DILLEN RME AND VAN DER NOORDAA J. (1990). Rapid and simple method for purification of nucleic acids. *J. Clin. Microbiol.*, **28**, 495–503.

DAS BC, SHARMA JK, GOPALKRISHNA V, CAS DK, SINGH V, GISSMANN L, ZUR HAUSEN H AND LUTHRA UK. (1992). A high frequency of human papillomavirus DNA sequences in cervical carcinomas of Indian women as revealed by Southern blot hybridization and polymerase chain reaction. *J. Med. Virol.*, **36**, 239–245.

DE RODA HUSMAN AM, WALBOOMERS JMM, MEIJER CJLM, RISSE EKJ, SCHIPPER MEI, HELMERHORST TM, BLEKER OP, DELIUS H, VAN DEN BRULE AJC AND SNIJDERS PJF. (1994). Analysis of cytomorphologically abnormal cervical scrapes for the presence of 27 mucosotropic human papillomavirus genotypes using polymerase chain reaction. *Int. J. Cancer*, **56**, 802–806.

DE RODA HUSMAN AM, WALBOOMERS JMM, VAN DEN BRULE AJC, MEIJER CJLM AND SNIJDERS PJF. (1995). The use of general primers GP5 and GP6 elongated at their 3′ ends with adjacent highly conserved sequences improves human papillomavirus detection by polymerase chain reaction. *J. Gen. Virol.*, (in press).

EISENSTEIN BI. (1990). The polymerase chain reaction: a new method of using molecular genetics for medical diagnosis. *N. Engl. J. Med.*, **322**, 178–183.

GALL K, PAVIČIĆ D, PAVELIĆ J, AUDY-JURKOVIĆ S AND PAVELIĆ K. (1993). PCR amplification from stained cytological smears. *J. Clin. Pathol.*, **46**, 378–379.

GOELZ SE, HAMILTON SR AND VOGELSTEIN B. (1985). Purification of DNA from formaldehyde-fixed and paraffin embedded human tissue. *Biochem. Biophys. Res. Commun.*, **130**, 118–126.

GRÉGOIRE L, ARELLA M, CAMPIONE-PICCARDO J AND LANCASTER WD. (1989). Amplification of human papillomavirus DNA sequences by using conserved primers. *J. Clin. Microbiol.* **27**, 2660–2665.

HIGUCHI R. (1990). Rapid, efficient DNA extraction for PCR from cells and blood. *Perkin Elmer Cetus Amplifications*, **2**, 1–3.

JACKSON DP, BELL S, PAYNE J, LEWIS FA, SUTTON J, TAYLOR GR AND QUIRKE P. (1989). Extraction and amplification of DNA from archival haematoxylin and eosin sections and cervical cytology Papanicolaou smears. *Nucleic Acids Res.*, **17**, 10,134.

LUNGU O, SUN XW, FELIX J, RICHART RM, SILVERSTEIN S AND WRIGHT TC. (1992). Relationship of human papillomavirus type to grade of cervical intraepithelial neoplasia. *JAMA*, **267**, 2493–2496.

MANOS MM, TING Y, WRIGHT DK, LEWIS AJ, BROKER TR AND WOLINSKY SM. (1989). Use of polymerase chain reaction amplification for the detection of genital human papillomaviruses. *Cancer cells*, **7**: 209–214.

O'LEARY JJ, BROWNE G, JOHNSON MI, LANDERS RJ, CROWLEY M, HEALY I, STREET JT, POLLOCK AM, LEWIS FA, ANDREW A, CULLINANE C, MOHAMDEE O, KEALY WF, HOGAN J AND DOYLE CT. (1994). PCR in situ hybridisation detection of HPV 16 in fixed CaSki and fixed SiHa cell lines. *J. Clin. Pathol.*, **47**, 933–938.

RAKOCZY P, STERRETT G, KULSKI J, WHITAKER D, HUTCHINSON L, MACKENZIE J AND PIXLEY E. (1990). Time trends in the prevalence of human papillomavirus infections in archival Papanicolaou smears: analysis by cytology, DNA hybridization and polymerase chain reaction. *J. Med. Virol.*, **32**, 10–17.

RESNICK RM, CORNELISSEN MTE, WRIGHT DK, EICHINGER GH, FOX HS, TER SCHEGGET J AND MANOS MM. (1990). Detection and typing of human papillomavirus in archival cervical cancer specimens by DNA amplification with consensus primers. *J. Natl Cancer Inst.*, **82**, 1477–1484.

SAIKI RK, SCHARF S, FALOONA F, MULLIS KB, HORN GT, ERLICH HA AND ARNHEIM N. (1985). Enzymatic amplification of beta-globin genomic sequences and restriction site analysis for diagnosis of sickle-cell anemia. *Science*, **230**, 1350–1354.

SAIKI RK, GELFAND DH, STOFFEL S, SCHARF SJ, HIGUCHI R, HORN GT, MULLIS KB AND ERLICH HA. (1988). Primer-directed enzymatic amplification of DNA with a thermostable DNA polymerase. *Science*, **239**, 487–494.

SHIBATA DK, ARNHÈIM N AND MARTIN WJ. (1988). Detection of human papillomavirus in paraffin-embedded tissue using the polymerase chain reaction. *J. Exp. Med.*, **167**, 225–230.

SLEBOS RJC, HRUBAN RH, DALESIO O, MOOI WJ, OFFERHAUS GJA AND RODENHUIS S. (1991). Relationship between K-ras oncogene activation and smoking in adenocarcinoma of the human lung. *J. Natl Cancer Inst.*, **83**, 1024–1027.

SMITS HL, TIEBEN LM, TJONG-A-HUNG SP, JEBBINK MF, MINNAAR RP, JANSEN CL AND TER SCHEGGET J. (1992). Detection and typing of human papillomaviruses present in fixed and stained archival cervical smears by a consensus polymerase chain reaction and direct sequence analysis allow the identification of a broad spectrum of human papillomavirus types. *J. Gen. Virol.*, **73**, 3263–3268.

SNIJDERS PJF, VAN DEN BRULE AJC, SCHRIJNEMAKERS HFJ, SNOW G, MEIJER CJLM AND WALBOOMERS JMM. (1990). The use of general primers in the polymerase chain reaction permits the detection of a broad spectrum of human papillomavirus genotypes. *J. Gen. Virol.*, **71**, 173–181.

SNIJDERS PJF, MEIJER CJLM AND WALBOAOMERS JMM. (1991). Degenerate primers based on highly conserved regions of amino acid sequence in papillomaviruses can be used in a generalized polymerase chain reaction to detect productive human papillomavirus infections. *J. Gen. Virol.*, **72**, 2781–2786.

VAN DEN BRULE AJC, MEIJER CJLM, BAKELS V, KENEMANS P AND WALBOOMERS JMM. (1990). Rapid detection of human papillomavirus in cervical scrapes by combined general primer-mediated and type-specific polymerase chain reaction. *J. Clin. Microbiol.*, **28**, 2739–2743.

VAN DEN BRULE AJC, WALBOOMERS JMM, DU MAINE M, KENEMANS P AND MEIJER CJLM. (1991). Difference in prevalence of human papillomavirus genotypes in cytomorphologically normal cervical smears is associated with a history of cervical intraepithelial neoplasia. *Int. J. Cancer*, **48**, 404–408.

WALBOOMERS JMM, MELCHERS WJG, MULLINK H, MEIJER CJLM, STRUYK A, QUINT WGJ, VAN DER NOORDAA J AND TER SCHEGGET J. (1988). Sensitivity of in situ detection with biotinylated probes of human papillomavirus type 16 DNA in frozen tissue sections of squamous cell carcinomas of the cervix. *Am. J. Pathol.*, **131**, 587–594.

WALBOOMERS JMM, VAN DEN BRULE AJC, SNIJDERS PJF AND MEIJER CJLM. (1992). The polymerase chain reaction for human papillomavirus screening in diagnostic cytopathology of the cervix. In *Diagnostic Molecular Pathology: A practical Approach*, Vol. II, Herrington CS and McGee JOD (eds) pp. 153–172. Oxford University Press: New York.

WALBOOMERS JMM, DE RODA HUSMAN AM, VAN DEN BRULE AJC, SNIJDERS PJF AND MEIJER CJLM. (1994). Detection of genital human papillomavirus infections: critical review of methods and prevalence studies in relation to cervical cancer. In *Human Papillomaviruses and Cerival Cancer*, Stern PL and Stanley M (eds) pp. 41–71. Oxford University Press: New York.

WALBOOMERS JMM, DE RODA HUSMAN AM, SNIJDERS PJF, STEL HV, RISSE EKJ, HELMERHORST ThJM, VOORHORST FJ AND MEIJER CJLM. (1995). The presence of human papillomavirus in cytomorphologically false negative archival cervical smears: results of a retrospective analysis. *J. Clin. Pathol.* (in press).